# A Comparison of Morphology among Four Termite Species with Different Moisture Requirements

**DOI:** 10.3390/insects11050262

**Published:** 2020-04-25

**Authors:** John Zukowski, Nan-Yao Su

**Affiliations:** 1 Department of Sciences, John Jay College of Criminal Justice, City University of New York, 524 West 59th Street, New York, NY 10019, USA; 2 Ft. Lauderdale Research and Education Center, Department of Entomology and Nematology, Institute of Food and Agricultural Sciences, University of Florida, 3205 College Ave., Ft. Lauderdale, FL 33314, USA; nysu@ufl.edu

**Keywords:** cuticle, dampwood termite, drywood termite, rectal pad, spiracle, subterranean termite

## Abstract

The thicknesses of the cuticle and rectal pads, and the spiracle morphology were compared for four termite species from different habitats, including one drywood termite, *Cryptotermes brevis* Walker, one “wetwood” termite, *Cryptotermes cavifrons* Banks, one subterranean termite, *Coptotermes formosanus* Shiraki, and one dampwood termite, *Neotermes jouteli* (Banks). Cuticle thicknesses were significantly different among all four termite species. *Neotermes jouteli* had the thickest cuticle, while *Co. formosanus* had the thinnest. The cuticle of *C. brevis* was thicker than that of *C. cavifrons* and *Co. formosanus*, which may reflect a comparably greater need to prevent water loss in drier habitats for *C. brevis*. Rectal pad widths were significantly different among all four termite species, except those of *C. brevis* with *N. jouteli*. The rectal pads of *N. jouteli* and *C. brevis* were thicker than those of *C. cavifrons* and *Co. formosanus*, and the rectal pads of *C. cavifrons* were thicker than those of *Co. formosanus* in turn. Larger rectal pads likely account for the water conservation mechanism of producing dry, pelleted frass in the kalotermitids (*N. jouteli*, *C. brevis*, and *C. cavifrons*). Morphological observations of the spiracles showed the presence of protuberances (atrial arms) in the three kalotermitids. The function of this protuberance is unclear, but it may serve as a sac-like structure, aiding in gas exchange, or a moisture trap aiding in the prevention of water loss through evaporation.

## 1. Introduction

Due to their small size and relatively unsclerotized body, water loss is an important problem facing all termite species [1]. In order to keep a termite colony healthy, individuals must conserve their water resources. Termites generally live in confined galleries and nesting structures and maintain the relative humidity (RH) of the nest and foraging tunnels at desirable levels to prevent severe desiccation. Body water is lost via evaporation through the cuticle, buccal and anal openings, and spiracles [1,2]. The cuticle, the major interface involved in body water loss, includes microstructures for aid in the prevention of this loss (i.e., cement and wax layers). Body water is also lost from the spiracles during respiration and involves loss as water vapor associated with respirative gas exchange. During normal respiration when the spiracles are open, water vapor escapes with gaseous CO_2_.

Fecal deposition is yet another way water is lost and termites of the family Kalotermitidae are known to reabsorb water from their feces as it passes through the rectum, resulting in hard, dry, pelleted frass that is a distinctive sign of a drywood termite infestation. Termites also acquire water resources metabolically from the wood they live in and feed on. This is a characteristic of termites utilizing dry wood because accesses to direct water resources are so infrequent in their habitats. Kalotermitid dampwood termites also deposit pelleted frass, but the feces soften and form clumps due to the moisture conditions of the habitat. Subterranean termites often use their more liquid feces in the construction of carton and in the repair of the nests and galleries. The distinct types of frass are mainly due to differences in rectal pad physiology and differences in the rectums of termites have been documented [3].

The four termite species examined in this study live in relatively different microhabitats. Although of the same genus, *Cryptotermes cavifrons* Banks (“wetwood” termite) is found inhabiting relatively wet or dry wood in natural environments, whereas *Cryptotermes brevis* Walker (drywood termite) is almost always found in dry wood (lumber) of structures. Colonies of the Formosan subterranean termite, *Coptotermes formosanus* Shiraki (subterranean termite) are found in very humid underground galleries and tunnels, whereas *Neotermes jouteli* (Banks) (dampwood termite) lives in the high-humidity environment of wet, often dead, tree branches. All four species in this study were collected from the generally humid environment of south Florida, which may reduce the differences seen in their respective habitats. Considering the various habitats in which these termites are found, we hypothesized that those species that live in drier habitats will have thicker rectal pads and cuticles and likely exhibit a difference in spiracle morphology. To examine this, we compared the differences in the thickness of the cuticles and rectal pads, and the spiracle morphology among these four termite species.

## 2. Materials and Methods

### 2.1. Termite Collection

Individuals from colonies of *C. brevis*, *C. cavifrons*, *Co. formosanus*, and *N. jouteli* were collected in Broward County, Florida. The colonies of *C. brevis*, *C. cavifrons*, and *N. jouteli* were collected from infested pieces of wood. Colonies of *Co. formosanus* were collected from bucket traps, as described by Su and Scheffrahn [4]. All termites were kept in rectangular polystyrene boxes (17.15 by 12.22 by 6.03 cm) and stored in an incubator at 26.4 °C and 41.5% RH. *Coptotermes formosanus* and *N. jouteli* were kept with pieces of wood as food and shelter and were regularly misted with water to maintain >95% RH. Both *Cryptotermes* termite species were kept with pieces of wood as food and shelter, but only *C. cavifrons* were provided with small dishes of water that were regularly refilled. The populations of each termite species survived well in these conditions and were used for experiments as needed. Termites were kept in the incubator for no more than six months before use.

### 2.2. Sectioning, Staining, and Slide Preparation

Individual workers or pseudergates (six per species) were randomly sampled from populations for dissection and sectioning. The characteristics of the cuticle, rectal pads, and spiracles of the four termite species were examined. Termite specimens were prepared for histological sectioning by removing the heads and legs. The specimens were then placed in Bouin’s fixative solution (75% aqueous picric acid, 20% formaldehyde, 5% acetic acid) for at least seven days [5]. They were then dehydrated for 30 min three times in successive solutions of 75% and 95% ethanol, for 30 min a single time in pure n-butanol, and for 24 h three times in pure n-butanol.

Following dehydration, specimens were passed through a single mixture of 50% paraffin wax and 50% n-butanol for 6 h at 60 °C, and three times in containers of pure paraffin wax for 24 h at 60 °C to replace the butanol with paraffin. Specimens were then embedded in blocks of pure paraffin wax, sectioned with a microtome (~7 µm sections), attached to slides, and stained with a modified Azan Heidenhaim protocol [6,7]. The stained slides (with cover slips and Permount mounting medium) were observed at varying magnification with a compound microscope (Leica DM500 B, Leica Microsystems GmbH, Wetzlar, Germany) coupled with a DFC450 Leica camera in order to measure the thickness of the cuticle and rectal pads. A dissecting microscope (Olympus SZX9, Olympus Corporation, Shinjuku, Tokyo, Japan) was used to observe the morphology of the spiracles. Abdominal sections were used to avoid complications with staining and sectioning more sclerotized parts of the termite body (i.e., head and thorax) and take advantage of the thinner more transparent integument therein.

Pictures of the stained abdominal sections were taken with Leica Application Suite (LAS) software and stored for measurement analysis with the GNU Image Manupulation Program (GIMP, The GIMP team, version 2.8.14, www.gimp.org, ^©^ 2020–2014). Measurement of the width of the cuticle was taken where staining had allowed for determination of the cuticle thickness at 200× magnification (i.e., cuticle was colored pink or purple). Care was taken to measure cuticle widths as close to perpendicular to the plane of the cuticle as possible and to avoid using sections of the cuticle that were twisted or otherwise in poor condition for analysis. Analysis of the rectal pads was made using the width of the pads observed through transverse sections of the abdomen. Care was also taken to measure rectal pad widths as close to perpendicular to the plane of the rectal pad as possible and to avoid using pads that were oblong or otherwise in poor condition for analysis. Multiple measurements of the rectal pads and cuticle were taken from multiple sections of each of the six termite individuals. Observation of the spiracles was made using the compound and light microscopes and analysis was solely observational and based on morphology.

### 2.3. Data Analysis

Statistical analysis was carried out using JMP statistical software (JMP^®^ Pro, 2013. version 11.0. SAS Institute Inc., Cary, NC, USA). An ANOVA (analysis of variance) was used to examine differences in overall cuticle thickness (micron) and rectal pad width (micron) among four termite species. Significant differences were separated using Tukey’s Honest Significant Difference (HSD) post hoc tests.

## 3. Results

### 3.1. Cuticle Thickness

**Figure 1:**
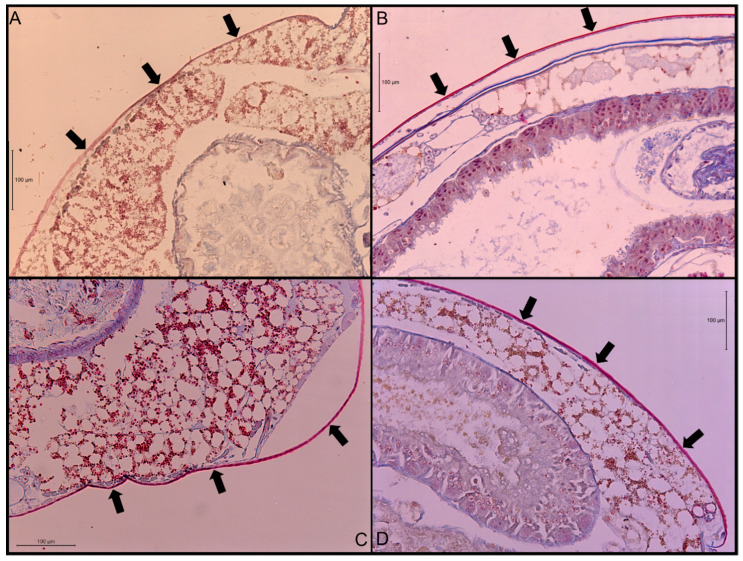
Transverse sections of stained abdominal cuticle at 200× magnification. (**A**) *Co. formosanus*; (**B**) *N. jouteli*; (**C**) *C. cavifrons*; (**D**) *C. brevis*. The arrows indicate the cuticle.

The ANOVA test indicated that there was a significant difference in cuticle thickness (df = 3, F = 55, *p*-value < 0.0001) among the four termite species. When separating differences using Tukey’s HSD, all four species were found to be significantly different, with *N. jouteli* having the thickest cuticle and *Co. formosanus* having the thinnest (Table 1). Multiple measurements were taken from six termite individuals per species. Cuticle thickness is generally uniform along the abdomen, except where it transitions to more flexible connective membrane. A visual example of a stained cuticle is shown in Figure 1.

### 3.2. Rectal Pad Width

**Figure 2:**
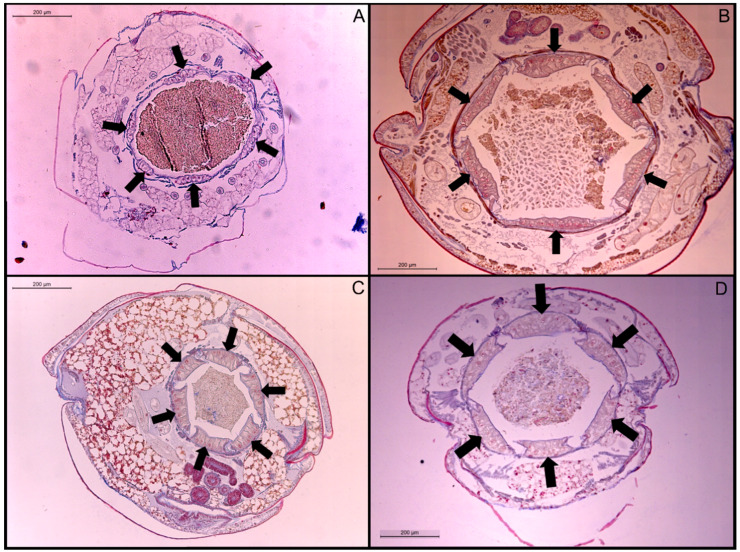
Transverse sections of stained rectal pads at 200× magnification. (**A**) *Co. formosanus*; (**B**) *N. jouteli*; (**C**) *C. cavifrons*; (**D**) *C. brevis*. The arrows indicate individual rectal pads.

The ANOVA test indicated that there was a significant difference in rectal pad width (df = 3, F = 48.4, *p*-value < 0.0001) among the four termite species. Rectal pad widths were not significantly different between *N. jouteli* and *C. brevis* (Table 2), but both these species had significantly thicker pads than *C. cavifrons* and *Co. formosanus*, with *Co. formosanus* having the thinnest rectal pads (Table 2). Multiple measurements were taken from six termite individuals per species. Visual examples of the rectal pads of the four termite species are shown in Figure 2. The rectal pads of *N. jouteli* and *C. brevis* were generally shorter and thicker, often taking a swollen, more bulbous shape.

### 3.3. Abdominal Spiracles

**Figure 3:**
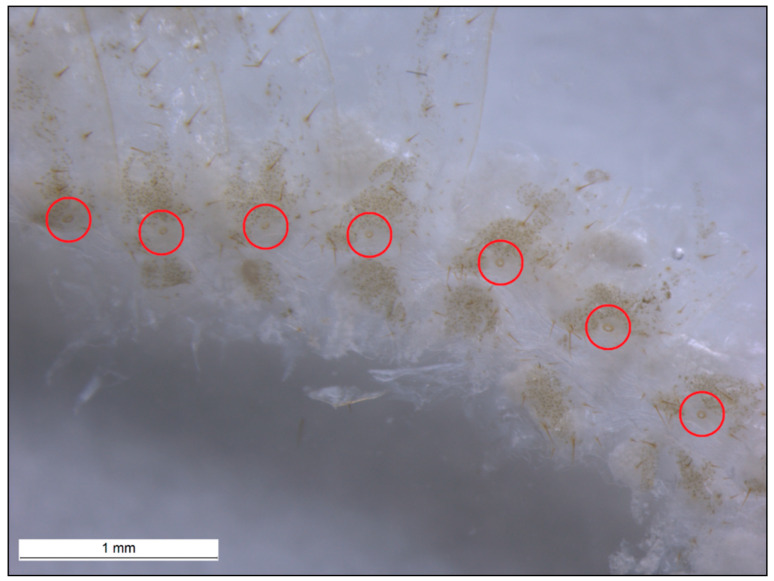
Abdominal spiracles marked with red circles on the tergites and near the pleural membrane of *N. jouteli*. Abdominal spiracles of *Co. formosanus*, *C. cavifrons*, and *C. brevis* were found in similar locations on their bodies.

**Figure 4:**
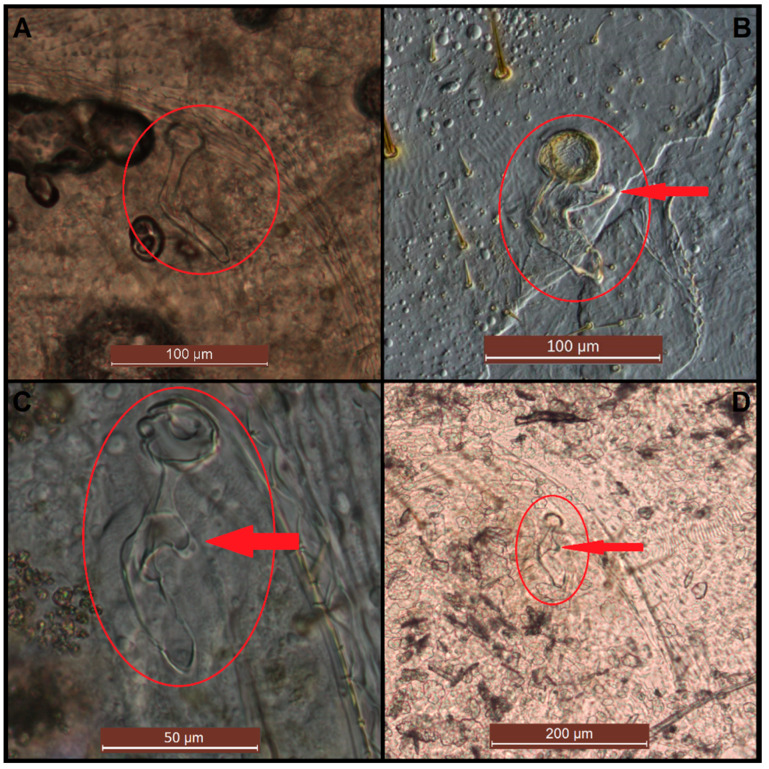
Dissected spiracles (red circles) at various magnifications. (**A**) *Co. formosanus*; (**B**) *N. jouteli*; (**C**) *C. cavifrons*; (**D**) *C. brevis*. The red arrows indicate spiracular protuberances (secondary atrial arms).

**Figure 5:**
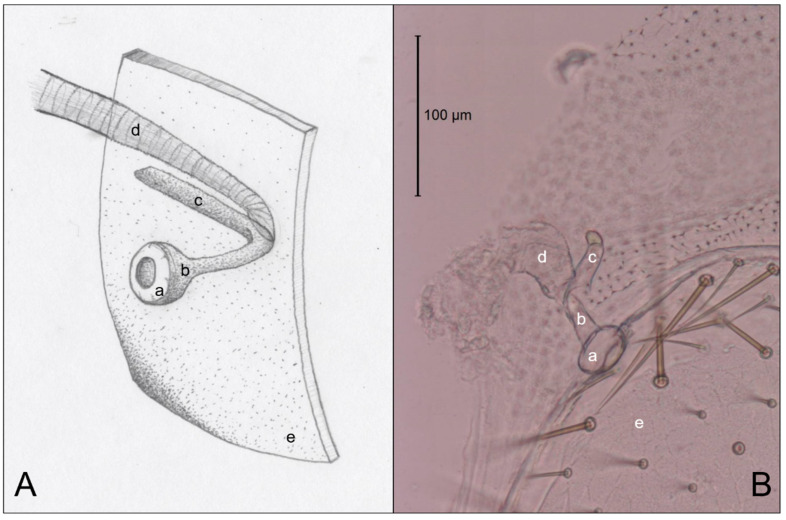
Comparison of an artist’s interpretation and photograph of the attachment of the spiracle structure and the trachea of *Co. formosanus*. (**A**) Drawing of spiracle-tracheal attachment; (**B**) Photograph illustrating spiracle-tracheal attachment. **a**: spiracle cap (peritreme); **b**: atrium; **c**: atrial arm of spiracle structure; **d**: tracheal trunk; **e**: cuticle.

**Figure 6:**
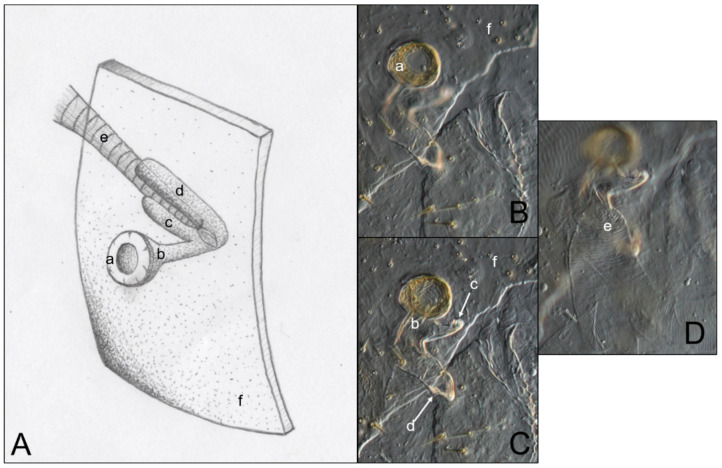
Comparison of an artist’s interpretation and photographs of the attachment of the spiracle structure and the trachea in three kalotermitid species (*C. brevis*, *C. cavifrons*, and *N. jouteli*). (**A**) Drawing of spiracle-tracheal attachment; (**B**–**D**) Photograph illustrating spiracular-tracheal attachment at different focal levels. **a**: spiracle cap (peritreme); **b**: atrium; **c**: atrial arm of spiracle structure; **d**: additional atrial arm of spiracle structure; **e**: tracheal trunk; **f**: cuticle.

Figure 3 is an example of the abdominal spiracles after the dissection of a specimen of *N. jouteli*. All four species had spiracles on each of the eight tergites of the abdomen, situated near the pleural membrane (as in Figure 3, red circles). Dissected spiracles can be seen through the transparent cuticle of the termite body. In *Co. formosanus*, the spiracle had a j-shape, but lacked the protuberance seen in the kalotermitids (Figure 4A). In *C. cavifrons*, *C. brevis*, and *N. jouteli*, the spiracles take on a j-shape, with an extension (atrial arm) protruding from the main channel of the spiracle (Figure 4B–D, red arrows). In *Co. formosanus*, the lack of the protuberance is evident (Figure 5A,B), as the tracheal trunk (Figure 5d) is attached to the underside of the main structural curve (i.e., the beginning of the tail of the j-shape), with a main atrial arm (Figure 5c), but no additional arm present. In the kalotermitids (exemplified in Figure 6A–D), however, the trachea (Figure 6e) appeared to connect beneath the structural curves, adjacent to the main atrial arm (Figure 6c) and an additional atrial arm (Figure 6d), the “protuberance.” The spiracular structure of *Co. formosanus* was generally longer and thinner than the structures found in the kalotermitids. Stained and sectioned specimens, in conjunction with unstained dissection slides helped visualize the morphology of the spiracular structures.

## 4. Discussion

### 4.1. Cuticle Thickness

The cuticle (and sclerotization) of insects is an important component of defense from external factors, as well as the stability of the internal and external milieu. The permeability of the termite cuticle to water is key to this stability and resistance to desiccation. A general lack of sclerotization in termites would seem to compound their difficulty with preventing water loss. While examining the cuticle, the assumption was that a thicker cuticle contributes to a lower percentage of body water lost to evaporation over a given amount of time. This would be reflected in lower cuticular permeability (CP) values. CP is defined as the amount of water lost (μg) per unit surface area (cm^2^) per unit time (h) per unit saturation deficit (mmHg), and is often used to characterize and compare evaporative water loss from the cuticle of insects and other arthropods [1,8]. This assumption regarding cuticle thickness is not supported when examining previous work on CP in beetles and cockroaches [9,10,11,12,13,14]. Even more heavily sclerotized insects can lose water easily, as reflected in relatively high CP values in these studies. Locke [15], Beament [16], Wigglesworth [8], and Ebeling and Wagner [17] found that treatment of the insect cuticle with wax layer disruptants (i.e., peanut oil, abrasive dusts) and sorptive dusts caused the rapid loss of body water (desiccation). Similar results were seen in studies on the cuticle of termites by Collins [18] and Sponsler and Appel [19]. Considered together, these studies indicated that cuticle thickness is not as important as its composition (layering and hydrocarbons), with a lipid layer acting as the main barrier to water loss [20]. The same is likely true for termites, and efforts should be made to analyze the layering components and surface hydrocarbons of the cuticles of termite species from various habitats, such as those reported here and others. The results from this study and a previous study [21] indicate that physiological traits, such as rectal pad width and spiracle morphology, could serve as predictors of desiccation resistance in termite species. Cuticle thickness is likely not a good predictor, but the composition and/or thickness of the wax layer component might be.

The thin cuticle of *Co. formosanus* reported here may account (in part) for the observation that this species desiccates quickly in dry to moderately moist conditions [21]. A thin, non-sclerotized cuticle does not allow for the efficient trapping of body water within a termite. Termites such as *Co. formosanus* lack the ability to prevent water from evaporating through the cuticle to an appreciable degree, which leads to rapid desiccation and death. This is a major reason why subterranean termites rely heavily on behavioral mechanisms and the modification of their microhabitat to prevent desiccation. High CP values, then, would be advantageous for producing and utilizing these modifications and mechanisms. *Cryptotermes brevis* and *N. jouteli* had similarly greater cuticle thicknesses than *Co. formosanus* and *C. cavifrons*. Since *C. brevis* lives in dry habitats, the thick cuticle that includes a wax layer barrier allows this species to hold water within the body in a desiccating environment [16]. In contrast, *N. jouteli* lives in an environment of damp wood, so a thicker cuticle does not seem necessary. However, since *N. jouteli* is a larger species, the thicker cuticle may relate to its body size/volume (i.e., to maintain structural integrity) or prevent water absorption from disrupting physiological processes (“water logging”). *C. cavifrons* had intermediate cuticle thicknesses, thicker than *Co. formosanus* and thinner than *C. brevis* and *N. jouteli*. *Cryptotermes cavifrons* can be found living in wood with both low and high moisture content in a natural habitat. Inherent in this habitat are fluctuations in humidity and moisture availability, as well as possible disturbances to their single-piece food source. They should, therefore, not only be equipped to tolerate the changes in the conditions of their environment, but also be equipped to adequately prevent water from escaping the body in normal and stressed situations. Further analysis allowing for the identification of cuticle layers and thicknesses via scanning or transmission electron microscopy and/or staining and cuticular hydrocarbon analysis via gas chromatography–mass spectroscopy is warranted.

### 4.2. Rectal Pad Width

Rectal pads function to aid in the removal of water resources from faecal matter. *Cryptotermes brevis* individuals re-absorb most of the water from consumed wood through the six thick rectal pads and produced six-sided fecal pellets that contain very little moisture [3,18]. Fecal pellets of *N. jouteli*, however, are loose or clumped and can be moist [22], suggesting that the thick rectal pads of *N. jouteli* re-absorb water from consumed wood like those of *C. brevis* and produce pelleted frass that is secondarily hydrated via the conditions of the microhabitat. As with the cuticle thickness, this may also relate to the large body size of *N. jouteli* and required rates of water turnover in this species. Due to the larger volume of gut content, *N. jouteli* may need thicker rectal pads to absorb water from consumed wood to satisfy its physiological/digestive needs. *Coptotermes formosanus* lives in moist underground galleries and does not have to re-absorb and/or conserve water within them to the same degree as *C. brevis*, which may explain their thinner rectal pads. Further analysis allowing for the determination of rectal pad area and shape would be prudent.

### 4.3. Abdominal Spiracles

The morphology and possible functional mechanisms of the spiracles of the four termite species were examined. Spiracles are involved in respiration and link the outside environment with the internal milieu through sclerotized holes in the cuticle. They are another orifice (besides the buccal and anal cavities) through which water vapor can escape the body. The function of the protuberance (secondary atrial arm) found in *N. jouteli* and the *Cryptotermes* species is unclear, though it may function as a point for muscle attachment, be an extension of the atrium, a separate air sac-like structure aiding in gas exchange, or a moisture/humidity trap aiding in the prevention of evaporative water loss [23]. Muscle attachments were observed in the slides, but exactly how these muscles were connected and their role in closing the spiracles was unclear. The closing apparatuses were clearly of the internal variety in all four species, and the atria of the spiracles of insects have been noted to be long and tubular in some cases [23]. The spiracle structure observed was clearly connected to and separate from the trachea, as evidenced by the lack of taenidia within this structure and the clear taenidial rings seen in the tracheal trunk. Such a difference in spiracle structure (i.e., an additional atrial arm) may help explain the greater desiccation resistance seen in the three kalotermitid termite species when compared to the only rhinotermitid species in a previous study [21]. Further study on the ultrastructure of the spiracles (e.g., scanning electron microscopy) of these and other termites is warranted, but it is clear that there is a structural difference in the spiracles of the Kalotermitidae and Rhinotermitidae in this study.

## 5. Conclusions

The results indicate that there is a difference in the rectal pad widths (thickness) of the four termite species in this study, and that this difference lends itself to their ability to resist desiccation through the production and utilization of faeces. The results also indicate a difference in widths (thickness) of the termite cuticle, and while cuticle thickness may lend itself to their ability to resist desiccation, it is likely that this resistance is imparted primarily from the wax layer component of the cuticle. Observed differences in the internal structure and morphology of the spiracles between the termite families (Rhinotermitidae and Kalotermitidae) may also aid in a differential ability to physiologically control resistance to desiccation.

## Figures and Tables

**Table 1:** Cuticle thicknesses (mean ± SEM) of four termite species.

Species	Cuticle Thickness (Microns) *	Observations
*C. brevis*	2.28 ± 0.03a	180
*C. cavifrons*	2.10 ± 0.03b	222
*Co. formosanus*	1.81 ± 0.03c	196
*N. jouteli*	2.51 ± 0.05d	339

* Values followed by the same letters are not significantly different (Tukey’s HSD at ɑ = 0.05).

**Table 2:** Rectal pad widths (mean ± SEM) of four termite species.

Species	Rectal Pad Widths (Microns) *	Observations
*C. brevis*	160.5 ± 4.2a	66
*C. cavifrons*	142.6 ± 3.7b	59
*Co. formosanus*	101.8 ± 4.8c	62
*N. jouteli*	171.6 ± 4.8a	55

* Values followed by the same letters are not significantly different (Tukey’s HSD at ɑ = 0.05).
